# Application of proteomics for improving crop protection/artificial regulation

**DOI:** 10.3389/fpls.2013.00522

**Published:** 2013-12-19

**Authors:** Setsuko Komatsu, Hans-Peter Mock, Pingfang Yang, Birte Svensson

**Affiliations:** ^1^National Institute of Crop ScienceTsukuba, Japan; ^2^Leibniz Institute of Plant Genetics and Crop Plant ResearchGatersleben, Germany; ^3^Key Laboratory of Plant Germplasm Enhancement and Specialty Agriculture, Wuhan Botanical Garden, Chinese Academy of SciencesWuhan, China; ^4^Enzyme and Protein Chemistry, Department of Systems Biology, Technical University of DenmarkLyngby, Denmark

**Keywords:** proteomics, crop production, protection, artificial regulation, agriculture

## Current status and challenges in crop proteomics

The application of proteomics for analyses of crop plants has rapidly increased within the last decade. Although proteomic techniques are routinely used in plant laboratories worldwide, and constitute powerful study tools, there is still considerable room for improvement. In particular, the fraction of the plant proteome that can be detected using current approaches is markedly lower than that of other “Omics” techniques and therefore does not give a complete representation of the cellular proteins.

In the majority of cited papers, two-dimensional electrophoresis (2-DE) gel is the predominant technique used for separating proteins. However, liquid chromatography (LC)-based proteome analysis is becoming increasingly common in many laboratories. Both protein separation techniques have specific advantages. With a standard 2-DE approach, protein modification and degradation can be rapidly visualized, whereas LC-based methods require much lower amounts of starting material. The application of crop proteomics has been hindered by the limited availability of genomic information. However, with the successful development of “next-generation” sequencing technologies, identification and annotation of proteins and their isoforms in a particular crop species is becoming much more straightforward.

A specific advantage of proteomics over other “Omics” techniques is the capacity to reveal post-translational modifications (PTMs), which is a prerequisite to determine the functional impact of protein modification on crop plant productivity. To date, about 300 PTMs have been identified through proteomic analyses. However, major efforts are needed to establish reliable tools and strategies for evaluating the impact of this increasing number of different PTMs in crops. Finally, crop proteomics is expected to become an essential part of integrated “Omics” approaches. However, a major challenge for crop proteomics will be keeping pace with the throughput capacity of other “Omics” techniques. The application of proteomics for the functional analysis of plants will benefit from advances in plant phenotyping. Specifically, improved techniques for the automated, non-invasive phenotyping of plant collections will assist in the selection of appropriate genotypes for proteomics-based functional analyses aimed at characterizing the relevant traits for future crop breeding.

## Progress in crop proteomics for artificial regulation

Proteomic studies have identified numerous proteins that play crucial roles in plant growth and development. However, determining how this wealth of information can be applied toward agriculture and the artificial regulation of crops is a major challenge. Seeds are one of the most important factors in crop production, as seed viability is related to crop yields. He and Yang (2013) applied proteomics to the study of the regulation of rice seed germination and showed that starch is degraded in endosperm and later biosynthesized in the embryo during germination, a process that appears to promote the gradual utilization of nutritional reserves.

Heterosis has been widely used in crop production, in which a sterile male line is critical for hybrid breeding. Identifying the proteins involved in the regulation of male sterility represents a major target in crop proteomic studies (Wang et al., 2013). In contrast to traditional breeding methods, the application of transgenic techniques is becoming increasingly popular to rapidly obtain crops with desired qualities. Evaluation of these genetically modified crops with proteomic methods is essential (Gong and Wang, 2013).

Due to impending changes in the global climate and continued industrialization, maintaining food safety represents a serious challenge worldwide. To sustainably feed the world population, effective methods to increase the efficiency of sunlight conversion are needed (Driever and Kromdijk, 2013). C4 plants are more efficient at light conversion than C3 plants because they contain two different types of chloroplasts. Comparative proteomic analyses of C4 chloroplasts (Zhao et al., 2013) might help to determine the key components that influence the efficiency of sunlight conversion (Manandhar-Shrestha et al., 2013).

The interaction between crops and other organisms is an important factor that influences the growth and eventual yield of crops. For example, the pathogen *Fusarium graminearum* causes head blight of small grain cereals and dramatically reduces grain yield and quality, which has great economic impact on the cereal industry. Proteomic analysis is expected to complement traditional molecular genetics approaches for studying the mechanisms by which this pathogen attacks cereal crops (Yang et al., 2013). The use of proteomics for analyzing the interaction between crops and bacteria, particularly the symbiotic interactions in legume root nodules (Salavati et al., 2013), has also been demonstrated (Afroz et al., 2013).

Most studies have been conducted on whole organs or tissues, which do not allow for the collection of spatial information. It is therefore expected that the use of MS imaging techniques, which have been successfully applied in the field of medicine, will aid in obtaining information on the spatial distribution of metabolites and proteins (Matros and Mock, 2013).

## Progress in crop proteomics for stress responses

Stress is a key limiting factor that impairs the growth and yield of agricultural crops. Stressful conditions often lead to delayed seed germination, reduced plant growth, and decreased crop yield. Proteins associated with the primary function of an organ are specifically accumulated in that organ/tissue or organelle. Komatsu and Hossain (2013) highlighted the need for organ-specific proteomic analyses to identify proteins that are commonly accumulated in organs under a wide range of abiotic stresses (Komatsu and Hossain, 2013). Furthermore, due to the nature of abiotic stress, intracellular compartments play a dominant role in plant stress responses. Nouri and Komatsu (2013) reported that a number of subcellularly localized proteins, including ion/water transporters, reactive oxygen species scavengers, and proteins related to signaling and transcriptional regulation, are involved in stress tolerance.

Jacoby et al. (2013) described the application of the emerging proteomic technology of multiplexed selective-reaction monitoring MS, which has increased accuracy and throughput, for enhancing these approaches and providing a clear method to rank the relative importance of the growing cohort of stress-responsive proteins. In addition to crops, proteomic techniques have been applied to the study of moss species that serve as model systems in plant science (Wang et al., 2013) and several agriculturally important fruits (Chan, 2013) under abiotic and biotic stresses. It was revealed that proteins involved in different metabolic pathways in fruit were activated after postharvest treatments, suggesting that biologists should focus on combination treatments to reduce postharvest decay for minimizing production losses (Chan, 2013). For moss, comparison of the stress responses between different treatments revealed that there is a closer relationship between abscisic acid (ABA) and salt or dehydration than there is between ABA and cold (Wang et al., 2012).

Abiotic stresses are major constraints facing global crop production. For example, high temperature impedes the development and growth of crops. Proteomic studies have revealed that activation of amylolytic enzymes by high temperature is a crucial trigger for grain chalkiness (Mitsui et al., 2013). Takahashi et al. (2013) examined responses to freezing stress, which causes serious problems for agricultural management, and found that the plasma membrane plays significant roles in signal perception and cellular homeostasis, indicating that plasma membrane proteins are the most important factors in determining the environmental stress tolerance of plants. Salt stress severely decreases crop production and growth; however, certain crop cultivars show significant tolerance against the negative effects of salinity. Many salt-responsive proteins have been detected in major crops and are thought to increase resistance to salt stress (Aghaei and Komatsu, 2013). Hossain and Komatsu (2013) described the recent contributions of proteomic studies toward the understanding of heavy metal stress responses in plants, particularly the use of redox proteomic approaches for studying heavy metal-induced protein oxidation. The findings presented in the above review article may shed light on the cross talk that appears to occur between different stress signal pathways. The application of proteomic approaches to collect such data will aid in the design of genetically engineered stress-tolerant crop plants.

## Outlook

With impending climate changes, a rapidly growing global population that is predicted to exceed 9 billion people within three decades, and increasing need for natural resources, such as water and minerals, greater insights into the foundations of sustainable food production are needed to ensure efficient crop yields and applications. To achieve these objectives, novel tools for protecting crops against biotic and abiotic stresses and for unraveling the mechanisms underlying the development and vitality of seeds are required. “Omics” technologies continue to be promising tools for such explorations. As complete genomes are available for an increasing number of crop and model plants, systems biology or integrated “Omics” approaches will help to unravel the underlying mechanisms of complex plant traits, such as resistance to stresses, at a molecular level. The widespread application of quantitative proteomic techniques in combination with sophisticated imaging techniques for the identification and mapping of PTMs is expected to provide detailed understanding of protein regulation in complex biological networks. Such multidisciplinary strategies will also aid in the design of approaches for mitigating the damaging effects of plant stressors and promoting beneficial plant–microbe interactions. Systems biology analysis will also help in the breeding of robust crop plants that are tolerant to environmental stresses and have high nutritional value. Future crop proteomic studies aimed at understanding the structural basis for the interactions between biological molecules will be critical for controlling the regulation and function of both crop and associated microbial proteins.
